# Digital twin virtual hospitals and rural health disparities: a six-country comparative study (2018–2024)

**DOI:** 10.3389/fpubh.2026.1741438

**Published:** 2026-04-08

**Authors:** Hualei Wang, Xiuhua Chai, Nan Zhao

**Affiliations:** 1 Hebei Children’s Hospital, Shijiazhuang, China; 2 Hebei Provincial Institute of Science and Technology Information, Shijiazhuang, China

**Keywords:** comparative study, digital twin, health equity, rural health, virtual hospitals

## Abstract

**Background:**

Rural health disparities are currently affecting 3.3 billion people worldwide. This study assesses the effectiveness of digital twin virtual hospitals in reducing rural health disparities.

**Methods:**

We examined health outcomes in six countries (United States, Australia, China, Brazil, India, Rwanda) between 2018 and 2024, employing fixed-effects panel models to evaluate the effect of digital health interventions on the Rural–Urban Mortality Ratio (RMR) and the gap in the management of chronic diseases.

**Results:**

The average reduction in rural/urban health inequality was 29.6%. For each unit of investment in digital health, the RMR was associated with a decrease of 2.14 percentage points (*p* < 0.001). Diminishing marginal returns occurred beyond the investment of $50 per capita. Lower-income countries achieved greater cost-efficiency, with Rwanda’s ROI of 1:5.2 compared to the United States’s 1:2.3. Three distinct implementation models were identified: technology-driven (United States, Australia), system integration (China), and leapfrog development (India, Rwanda).

**Conclusion:**

Digital twin virtual hospitals are associated with reductions in rural health disparities, potentially through predictive and personalized health strategies. Findings suggest that successful implementation depends more on contextually appropriate technology selection than on technological sophistication alone.

## 1 Introduction

Rural health inequalities represent a major challenge in global healthcare, adversely affecting over 3.3 billion people worldwide (1). According to the 2024 report published by the World Health Organization, there are 20–50% inequality differences between the health outcomes of the rural population compared with the health outcomes realized by the urban population across the world with respect to preventable deaths, the control of chronic illnesses, and the availability of health care (2). These inequalities undermine healthcare as a basic human right and threaten the “leave no one behind” commitment of Sustainable Development Goal 3.

The COVID-19 pandemic has also highlighted and widened the gap existing between the quality of health care provided in different areas (3, 4). A study published in the Lancet in 2021 revealed that the death rate from COVID-19 was 39% higher in the rural compared to the urban population, with some countries reporting 67% differences in mortality (5). There was the closure of 19 rural hospitals in the US due to the COVID-19 pandemic, the second wave in India contributed 70% of its cases from the rural areas, while the humanitarian crisis from the shortage of healthcare resources in the Amazon regions of Brazil left the world in shock (6–8).

However, crises can also catalyze innovation. Global investment in rural digital healthcare escalated from $12 billion in 2022 to $38 billion in 2023, with 2024 estimates projected at $58 billion based on first three quarters data. The remote coverage of the hospital populations increased from 300 million to 1.8 billion (9). New approaches, including the reform of the county medical community in China, the Ayushman Bharat Digital Mission in India, or the digitalization of community health workers in Rwanda, are some examples that show the potential of technology to reduce health inequalities (10–12). In particular, the technology of “digital twins” has the potential to trigger paradigm shifts in the health care of the rural population, thanks to its ability to implement predictive, precision, or “personalized” medicine by “mirroring” each human body individually (13–15).

Despite the encouraging outcomes of individual projects, there is currently a lack of systematic cross-country comparison of the outcomes of digital twin virtual hospital solutions. Existing cross-national digital health research has primarily focused on policy comparisons (2) or technology adoption patterns (9, 16), rather than quantitative evaluation of health equity outcomes. Most empirical studies remain confined to single countries or snapshot evaluations, leaving key questions unanswered: What is the effectiveness of the virtual hospital solution across countries with different levels of development? What is the optimal investment level? What are the crucial success factors? To what extent has the COVID-19 pandemic changed the course of the development of the digital health care system?

Based on the context, the proposed work will conduct an analysis on publicly available health data from the US, Australia, China, Brazil, India, and Rwanda from the period 2018 to 2024 with the following purposes: (1) quantitative evaluation of the effect size of the virtual hospital on health disparities, (2) exploring the models of implementation in different countries, (3) the cost-effectiveness analysis, and (4) developing policy recommendations on health inequalities according to the stage of development of the concerned countries.

In terms of innovation, this study is novel in three ways: the first longitudinal study on the entire wave of the COVID-19 pandemic, unlocking the role of crises in the digital health transformation (3), the first comparison study in six countries on the successful models of the digital twin health impacts, uncovering success from failures, and the provision of the first development stage Implementation Framework, offering the world the roadmap for spreading the solution. The three points, explored in this study, answer the contributions made by the study in relation to its application aimed at achieving “Health for All.”

## 2 Theoretical framework and literature review

### 2.1 Evolution of health equity theory in the digital age

Research on health equity has evolved from documenting disparities to understanding underlying mechanisms to evaluating interventions. This progression reflects (33) normative definition of health inequities as “avoidable, unnecessary, and unjust health differences.” A framework proposed by Solar and Irwin (34), developing the WHO definition on the social determinants of health, categorized the underlying mechanisms of health differences into structural, including “Socioeconomic status, Political,” or material, including “Behavioral, Factors, Health Systems” variables (17).

Nonetheless, these conventional approaches have neglected the importance of digital technology. Azzopardi-Muscat and Sørensen proposed the inclusion of digital literacy in the social determinants of health, contending “the digital divide is gradually emerging as an effect on health inequalities” for the first time in 2019 (5). The “Digital Health Equity Framework” was proposed by Crawford and Serhal (35), with four main components: “digital access, digital literacy, digital relevance, and digital trust” (18). The main implication is the technology itself is impartial, but its health influence relies on the process of embedding the technology in society.

The healthcare accessibility theory also needs revisioning. The classic five-dimensional construct proposed by Penchansky and Thomas (36), namely, availability, accessibility, affordability, acceptability, and accommodation, needs enlargement in the today’s electronic age context. Levesque et al. (37) proposed a sixth dimension, “perceivability,” emphasizing the identification of health needs (19). We further propose a seventh dimension, “operability,” implying the users’ operational efficiency with the electronic health resources, which is vital for health acceptance in the rural context of the electronic age (1, 20).

### 2.2 Digital twin technology: migration from industry to healthcare

The idea of the digital twin was developed within the aviation industry, with NASA developing virtual versions of their spacecraft to be used in diagnostics for the Apollo missions. A digital twin is described by Grieves (38): “Three-dimensional model consisting of the physical twin, the virtual twin, and logical links between the two.” The development of the digital twin was spurred on by the growth of Industry 4.0 (13).

The application of the concept of the digital twin in the health sector began in 2015, with the main focus on the creation of models of human organs, followed by the application of the technology in the field of surgery. The project “Heart Digital Twin” launched by Philips created precision in the diagnosis and treatment of heart diseases by processing imaging, physiology, and genetics information (21). The “Digital Patient Twin” developed by Siemens Healthineers expanded the application of the technology in the treatment of oncology patients with chronic diseases (22).

Initially limited to tertiary care settings, these applications had minimal impact on rural health. Population-scale digital twins represent a newer paradigm. Kamel Boulos et al. (39) described the notion of “Smart Healthy City Digital Twin,” consolidating environment, behavior, and health information for the management of population health (14). During the COVID-19 period, the city of Singapore used city-scale digital twins to model the spread of the epidemic to inform strategies for its control. The application of the paradigm within the context of resource-limited rural settings poses an issue due to lack of data, processing, or intellectual talent (15, 16).

Analysis of the literature indicates that there are three different levels of application of digital twins in healthcare, categorized based on their focus, with 45% application on precision medicine, 35% on hospital efficiency, and the last one involving the entire population, with 20% application (13, 14, 16). Rural application is extremely uncommon, with less than 5% involving purely theoretical discussion. However, emerging empirical evidence from low-resource rural settings demonstrates the feasibility of implementing digital health components that underpin digital twin systems. In Sub-Saharan Africa, Rwanda’s community health worker (CHW) mHealth program has shown significant improvements in maternal and newborn health service uptake through real-time data collection and feedback loops (12). Similar SMS-based interventions during COVID-19 demonstrated scalability for remote patient monitoring in resource-limited contexts. In South Asia, India’s eSanjeevani telemedicine platform completed over 100 million consultations by 2023, with rural areas accounting for approximately 60% of users, providing foundational infrastructure for more advanced digital twin applications (11). Studies from Brazil’s ConecteSUS initiative have documented improved chronic disease management in remote Amazon communities through integrated digital health records. While these interventions fall short of full digital twin implementation, they represent critical building blocks—real-time data mirroring, predictive risk stratification, and personalized feedback—that can evolve into comprehensive digital twin systems. This progression from basic mHealth to sophisticated digital twins forms the basis of the “leapfrog development” model examined in this study. Which is the service gap the proposed study plans to fill.

### 2.3 Global digital health policy landscape

A systematic review of health technology policies in six countries from 2018 to 2024 has revealed three different health technology policy paradigms.

The market-driven paradigm is represented by the US system. The 2020 Rural Digital Opportunity Fund allocated $20.4 billion to enhance broadband, while the 2021 Telehealth Modernization Act eased restrictions on practicing across state lines (6, 23). The policy aspects include the utilization of markets, with the government acting only as the facilitator/infrastructure developer. Strengths include vibrant innovation, with virtual hospital networks such as Mayo Clinic and Cleveland Clinic having the highest international quality, while the limitation is the lack of equitable geographical distribution, with insurance defining access to care.

The state-led pattern is represented by the example of China. The “Opinions on Promoting “Internet + Medical Health” Development” from 2018 had the purpose of state-led design, followed by the “Notice on Deepening the “Internet + Medical Health” Five-One Service Action” from 2020, referring to the execution process (10, 24). The administrative authority is the key to standardization, regional connection, and medical insurance connection. The county medical community is the organizational carrier of health informatics, realizing the “strong counties, active townships, and stable villages” for multi-level diagnosis and treatment, with strong efficiency but possibly restricted innovation capabilities.

The hybrid paradigm appears in the context of countries such as India, Brazil, or Rwanda. The Indian paradigm, AyushmanBharat, focuses on the provision of services to construct national health infrastructure by the government, with the involvement of the private health service providers (11, 25). The Brazilian paradigm, “ConecteSUS,” drives the inclusion of social capital in the integrated national health system (26, 27). The Rwandan paradigm receives technology investment, financial support, or resources from international cooperation, followed by the promotion of adapted apps once localization is completed (12, 28).

Analysis of healthcare policies shows the common success factors to be strategies with national focus, financial investment, payment system reform, or adjustment of healthcare regulations with flexibility, while challenges lie with the lack of standardization, standard privacy protection, quality surveillance requiring enhancement, or sustainability integrity concerns (2).

Empirical evaluations of these policies in rural contexts have yielded promising results. A systematic review of telehealth interventions in rural United States reported improved access and comparable outcomes to in-person care across multiple conditions (23). In Australia, the Royal Flying Doctor Service’s integration of telehealth reduced emergency evacuations by 15% in remote communities (29, 30). China’s county medical community pilots demonstrated 23% reduction in unnecessary referrals to urban tertiary hospitals (10, 24). These findings provide empirical grounding for the cross-national comparison undertaken in this study.

### 2.4 Comparative analysis of virtual hospital models

Cross-national comparative research on virtual hospitals and digital twin healthcare systems remains limited, though several recent studies have begun to address this gap. Vallée and Arutkin (9) provided a comprehensive review of virtual hospital implementations across Europe and North America, identifying significant variation in regulatory frameworks, reimbursement models, and technology adoption patterns. Elkefi and Asan (16) conducted a rapid literature review of digital twins for healthcare system management, noting that most implementations remained confined to single-institution or single-country contexts, with limited cross-border learning. A systematic scoping review by Maita et al. examined digital health solutions for rural health gaps across multiple countries, finding heterogeneous outcome measures that complicated direct comparisons (17, 20). Regarding low- and middle-income countries specifically, Ye et al. (10) reviewed China’s telehealth practices with implications for other developing nations, while Nakayama et al. (27) analyzed Brazil’s digital divide and barriers to telehealth equity. However, no study to date has systematically compared digital twin virtual hospital implementations across countries at different development levels or evaluated their differential impacts on rural health disparities. This represents a critical gap that the present study aims to address.

There is no common definition for virtual hospitals, with practices varying from one country to another. By the use of bibliometric analysis, we have categorized the different models of virtual hospital into four groups (9, 14). (1) Telemedicine Consultation Center Model, characterized by basic video consultations with low technology barriers; (2) Integrated Virtual Hospital System, providing comprehensive care across the entire treatment process; (3) Hybrid Reality Hospital, combining offline and online care delivery; and (4) Digital Twin Hospital Model, representing the most advanced form with predictive and personalized capabilities. Each model is described in detail below.

#### 2.4.1 Telemedicine consultation center model

The telemedicine consultation center model is basic for video consultations between the doctor and the patient, with lower technology barriers to entry but the ease of promotion being the only limitation to the service provided. The Royal Flying Doctor Service in Australia is one clear example, with satellite communication facilitating emergency consultations to remote locations (29, 30). This model is appropriate for sparsely populated areas with minimal or poor infrastructure.

#### 2.4.2 Integrated virtual hospital system

The integrated virtual hospital system provides consultation, examination, treatment, and follow-up care throughout the entire care process. The current system of internet hospitals in the context of China is able to realize the functionality close to the real one, relying on remote sharing of equipment, external testing, and medication transfer (10). The Mercy Virtual hospital in the US is the world’s largest virtual hospital, offering specialized care, including ICU care, remotely (23).

#### 2.4.3 Hybrid reality hospital

The hybrid reality hospital refers to the combination of offline and online care, with virtual hospitals being an extension of the hospital system itself. The Kaiser Permanente “Hospital at Home” care service provides patients with the benefits of hospital care from the comfort of their own homes, with the safety of the wearer device operation and periodic home visits (23).

#### 2.4.4 Digital twin hospital model

The hospital model provided by the digital twin is the most developed form of the future hospital paradigm. By developing digital twins of patients, hospitals, and populations, the model provides predictive and personalized medicine (13, 15, 16). “Future Hospital” pilot projects are currently underway in selected developed countries, including the pilot project underway in the Netherlands.

### 2.5 Conceptual model construction

According to the theoretical analysis and literature review, the study proposes a conceptual model of “Digital Twin Virtual Hospitals Promoting Rural Health Equity.” The proposed model consists of four levels:

The input level refers to basic conditions, which are described in relation to economic development, educational levels, and infrastructure, while policy inputs include sources of funding, legislation, and organizational structures.

The process level is the explanation of the mechanisms involved in the provision of health services through technology, including technology deployment, capacity building, and service provision, which encompass telemedicine, health management, and emergency response.

The level of output is concerned with direct outcomes, such as service utilization (consultation rate, compliance), health behaviors (self-management, preventive practices), or outcomes of care (disease management, mortality rate).

The level of the result focuses on final outcomes, that is, the consequences or the extent to which health equity is achieved, which is determined in relation to the reduction of the rural/urban gap, health outcomes in vulnerable populations, or health-related quality-of-life benefits.

The proposed model also highlights the crucial variables that play the moderating role, which include the acceptance of the culture, readiness, or the effect of sustainability on the outcomes of implementation. The proposed model assisted in the selection of variables to be studied in the analysis (5, 18, 31).

## 3 Methods

### 3.1 Study design

In particular, the study used longitudinal ecological design with the case study comparison method, analyzing publicly available health information from six countries between 2018 and 2024. The study had mixed methodology with the main approach being quantitative analysis supported by the review of policy documents. The study was classified into three clear periods: the baseline period before the pandemic was declared (2018–2019); the period of response to the pandemic (2020–2021); followed by the period of innovation in recovery from the pandemic from 2022 to 2024. This was because the world shifted toward the management of the COVID-19 pandemic from its pandemic stage to its endemic stage in early 2022, following the WHO announcement on March 11, 2020 (2, 3).

The study chose countries with a “most different systems” design, which maximized differences between countries with respect to levels of economic development, geography, health system structures, and maturity levels of information technology infrastructure. This design helps generate insights that reveal commonalities across differences, thus improving the external validity of the study’s findings.

### 3.2 Country selection and data sources

Six countries were purposively selected based on four criteria: availability of disaggregated health data, the implementation of health technology solutions in the target population, the inclusion of countries with different levels of income according to the World Bank, and the inclusion of countries with diverse health system structures. A total of six countries fulfilled these conditions.

The high-income countries are the US and Australia, with well-developed health surveillance systems but different models for the provision of health services in rural areas. The US system is market-driven but currently experiencing waves of hospital closure in the countryside (6), while the Australian model is one of universal provision, dealing with cases of geographical remoteness (29). The upper-middle-income countries are China, Brazil, and India, with a collective 40% of the world’s population, practicing the county medical community system (10), the unified health system (26), and the AyushmanBharat Digital Mission system, respectively. The Rwandan nation is the chosen country for the models developed due to resource limitation, utilizing leapfrog development in health informatics technology (12, 28).

Primary data were obtained from national health information databases between November 2024 and January 2025. Due to the time delay in national reporting, the annualized data from the first three quarters of 2024 was validated by the sensitivity analysis conducted on the entire data of 2023. The US data was obtained from the CDC WONDER database, the US HRSA Data Warehouse, while the Australian national, Aussie, data was taken from the AIHW, ABS databases, the Chinese from the National Health Commission Yearbooks, County Medical Community Monitoring System, while the Brazilian was from DATASUS, IBGE databases. Rwandan Data accessed via the DHIS2 system. Rural definition in each country was according to the national standard for statistical processing, namely, US—population density of less than 500 people per square mile, China—classification according to the NBSTAT, urban–rural, India—population number of less than 5,000, Australia—remoteness areas according to the ASGS, Brazil—rate of urbanization according to IBGE, Rwanda—administrative divisions. For comparability, sensitivity analyses recalculated using OECD unified standards (population density <150 people/square kilometer).

### 3.3 Variable definitions and measurements

The core result variable was the rural–urban gap in health, which was operationalized by three variables: the Rural Mortality Ratio (RMR), calculated by the age-standardized mortality rate in the rural areas divided by the mortality rate in the urban areas, the Preventable Hospitalization Gap (PHG), capturing the rate differences of ambulatory care sensitive conditions, and the Chronic Disease Management Effectiveness Gap (CDMEG), comparing the rate differences for the control of diabetes and hypertension (32).

The exposure variables are digital health investment (per capita spending, adjusted for purchasing power parity), virtual hospital reach, and the level of digital twin technology adoption (maturation stage, 1–5) (13, 14). The covariates include socioeconomic variables (GDP per capita, Gini ratio, educational attainment in years), health system variables (number of physicians per 100,000, number of beds per 100,000), and underlying digital components (internet usage, smartphone ownership) (5, 18, 27).

### 3.4 Statistical analysis

Descriptive analysis used means ± standard deviations and frequencies/percentages to summarize baseline characteristics, with temporal trends visualized using LOESS smoothing. Primary analysis employed two-way fixed effects panel data models:


$$Y_{\mathrm{it}}=\beta_{0}+\beta_{1}T_{\mathrm{it}}+\beta_{2}P2_{\mathrm{it}}+\beta_{3}P3_{\mathrm{it}}+\beta_{4}D_{\mathrm{it}}+\beta_{5}X_{\mathrm{it}}+\alpha_{i}+\gamma_{t}+\varepsilon_{\mathrm{it}}$$

Where *Y_it_* represents health disparity in country *i* at time *t*; *T_it_* is the linear time trend; *P2_it_* and *P3_it_* are period indicators for 2020–2021 and 2022–2024, respectively; *D_it_* represents digital health interventions; *X_it_* is the covariate vector; $\alpha_{i}$ and $\gamma_{t}$ are country and time fixed effects, respectively; $\varepsilon_{\mathrm{it}}$ is the error term.

To identify potential investment thresholds associated with diminishing marginal returns, we employed piecewise linear regression with unknown breakpoints. The optimal threshold was determined using the Davies test and iterative grid search method, testing candidate breakpoints across the observed investment range ($5–$450 per capita) at $5 intervals. The threshold yielding the lowest residual sum of squares was selected. To assess whether this threshold varied by development level, we conducted stratified analyses by World Bank income classification and tested interaction terms between investment and income group. Bootstrap resampling (1,000 iterations) was used to estimate 95% confidence intervals for the identified threshold.

Interrupted time series analysis evaluated COVID-19 impacts, setting interruption points at March 2020 and January 2022, using Newey-West standard errors to handle autocorrelation (3, 4). Sensitivity analyses included: alternative rural definitions, 5% Winsorization to exclude extreme values, MICE multiple imputation for missing data, Bootstrap resampling (1,000 iterations) for confidence interval estimation, and leave-one-country-out analysis.

Multicollinearity diagnostics showed all variable VIF values <5. Hausman test confirmed fixed effects model superiority over random effects (χ^2^ = 45.3, *p* < 0.001). Wooldridge test indicated first-order serial correlation (*F* = 12.4, *p* = 0.003), hence Driscoll-Kraay standard errors were applied for correction.

### 3.5 Cost-effectiveness and socioeconomic impact analysis

Cost-effectiveness analysis was conducted from the health system perspective. Two complementary metrics were calculated:

Return on Investment (ROI) was calculated as the ratio of monetized health benefits to digital health investment costs. Health benefits were monetized using country-specific estimates of productivity gains from reduced morbidity and mortality, valued at GDP per capita. Investment costs included government and private expenditure on digital health infrastructure, training, and operational expenses, obtained from national health accounts and supplementary reports.

Cost per Disability-Adjusted Life Year (DALY) averted was calculated as total digital health investment divided by DALYs averted. DALYs averted were estimated by comparing observed mortality and morbidity trends against counterfactual projections assuming no digital health intervention, using the baseline period (2018–2019) trajectory. Age-standardized disability weights were obtained from the Global Burden of Disease Study. Following WHO recommendations, interventions costing less than one times GDP per capita per DALY averted were considered highly cost-effective.

Socioeconomic impact analysis examined three secondary outcomes: (1) productivity impact, measured as change in average annual sick days in rural populations; (2) catastrophic health expenditure, defined as out-of-pocket health spending exceeding 40% of household non-food consumption; and (3) medical impoverishment rate, defined as the proportion of households pushed below national poverty lines due to health expenditures. Pre-post comparisons (2018 vs. 2024) were conducted with adjustment for baseline socioeconomic trends.

Sensitivity analyses tested the robustness of cost-effectiveness estimates to alternative discount rates (0, 3, 5%) and varying assumptions about intervention attribution (50, 75, 100% of observed improvements).

### 3.6 Quality control

Data quality assessment encompassed five dimensions: completeness, accuracy, timeliness, comparability, and accessibility, each scored 1–5, requiring mean score ≥3.5. Multi-source cross-validation ensured data reliability, with expert consultation resolving discrepancies exceeding 5%.

## 4 Results

### 4.1 Baseline characteristics and evolution of health disparities across six countries

Table 1 presents baseline characteristic comparisons across the six countries. In 2018, rural population proportions varied significantly, from 11% in Australia to 83% in Rwanda. Baseline Rural Mortality Ratios (RMR) indicated rural health disadvantages in all countries, with Rwanda highest (1.89) and the United States lowest (1.18). Rural–urban physician density ratios were universally below 0.4, with Rwanda at only 0.08. Digital infrastructure gaps were more pronounced in low-income countries, with Rwanda’s rural internet coverage 54 percentage points lower than urban areas (5, 19, 27).

**Table 1:** Baseline characteristics comparison across six countries (2018).

Indicator	United States	Australia	China	Brazil	India	Rwanda
Rural population (%)	14	11	41	13	65	83
GDP per capita (PPP, USD)	62,887	53,349	16,187	14,836	7,859	2,227
Baseline RMR	1.18	1.25	1.42	1.38	1.67	1.89
Physician density ratio (rural/urban)	0.31	0.28	0.35	0.22	0.18	0.08
Nurse density ratio (rural/urban)	0.42	0.45	0.48	0.38	0.31	0.26
Digital coverage gap (%)	−12	−18	−31	−26	−42	−54
Smartphone ownership gap (%)	−8	−11	−23	−19	−35	−47
Baseline digital health investment (USD/person)	23.4	31.2	8.7	5.3	2.1	0.8

**Figure 1:**
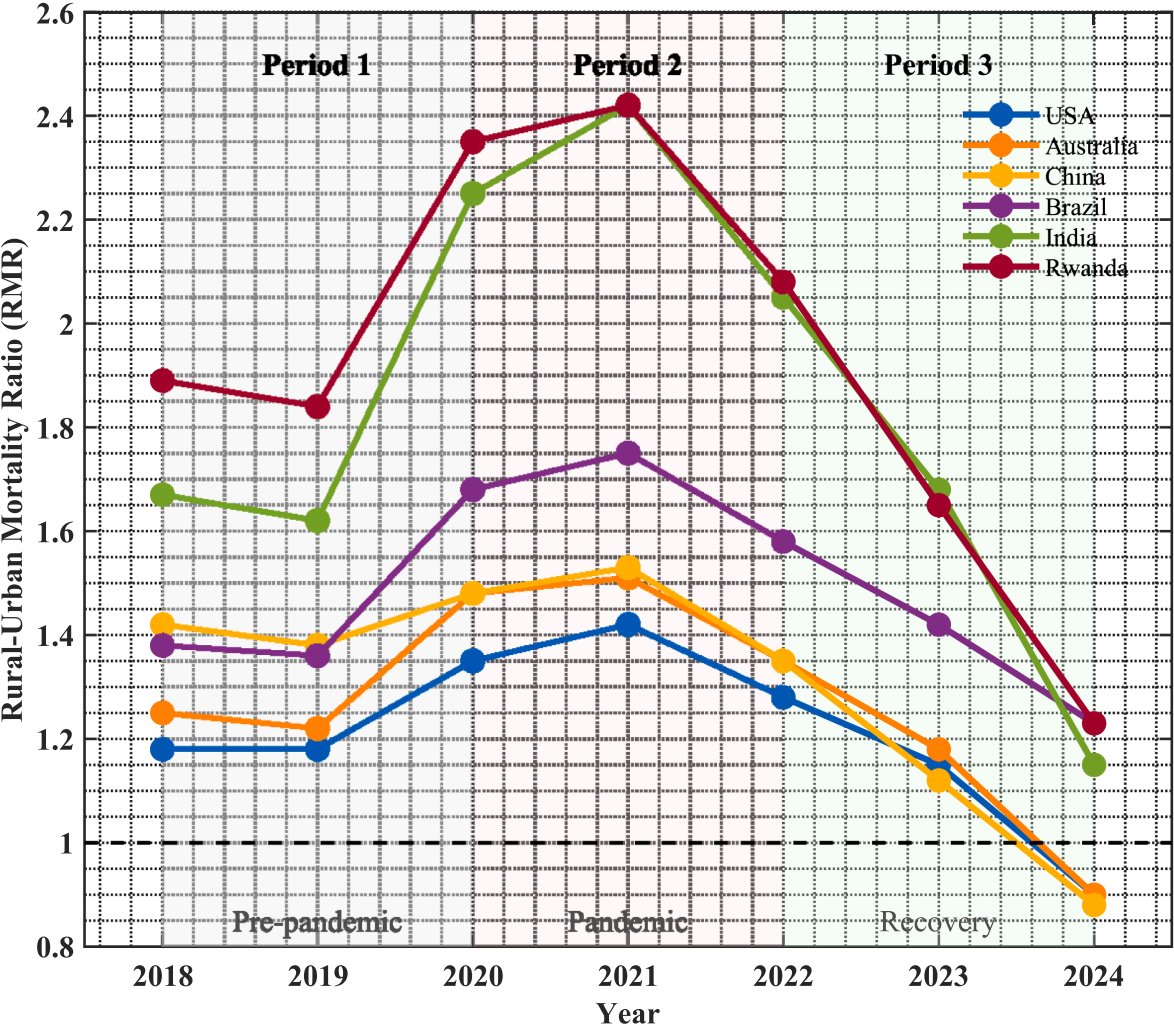
Evolution of rural–urban health disparities in six countries (2018–2024).

As shown in Figure 1, health disparities across the six countries exhibited three-phase evolution patterns from 2018 to 2024. During Phase 1 (2018–2019), health disparities improved slowly, with the six-country average RMR declining by 1.2% annually. Rwanda showed the fastest improvement (−2.8%/year), while the United States exhibited slight deterioration (+0.3%/year). Phase two (2020–2021) witnessed dramatic widening of health disparities due to the COVID-19 pandemic, with average RMR increasing 23.5% (3, 4). India suffered the most severe impact, with RMR rising from 1.67 to 2.42 (45% increase), while China remained relatively stable with only an 8% increase. Phase three (2022–2024) demonstrated significant correlation between rapid digital health deployment and health disparity improvement, with average RMR declining 18.6%, China showing the largest decrease (−31%) and Brazil the smallest (−11%).

This study defined digital twin hospitals as systems possessing three core functions: (1) real-time data mirroring—patient health data synchronized in real-time; (2) predictive simulation—health risk prediction based on historical data and algorithmic models; (3) optimized decision-making—personalized treatment recommendations (13–15). Systems with only teleconsultation capabilities were not classified as digital twins. Based on these criteria, as of 2024, only China (23 pilot counties) and the United States (7 health systems) had achieved true digital twin applications.

### 4.2 Deployment of digital health interventions

Between 2018 and 2024, digital health investment across the six countries grew exponentially. Cumulative per capita investment (PPP-adjusted) varied dramatically: United States $453 (6, 23), Australia $387 (29, 30), China $156 (10, 24), Brazil $89 (8, 26, 27), India $42 (11, 25), Rwanda $28 (12, 28). Investment growth rates negatively correlated with baseline development levels, with Rwanda and India exceeding 40% annual growth, while the United States and Australia averaged approximately 15%.

Virtual hospital coverage expanded from an average of 8.5% in 2019 to 58.3% in 2024 (9). Coverage expansion rates showed significant country variation: India grew 16-fold (3% → 48%), Rwanda 17.5-fold (2% → 35%), United States 5.6-fold (12% → 67%), Australia 4.3-fold (18% → 78%). China, despite a lower starting point (8%), reached 72% in 2024, covering the largest absolute rural population (296 million).

Digital twin technology application remained in early stages (13, 16). By 2024, only China and the United States reached Level 4 (scaled implementation), achieving digital twin-based chronic disease management and resource optimization in select regions. Australia and India were at Level 3 (pilot validation), Brazil at Level 2 (proof of concept), and Rwanda remained at Level 1 (exploratory phase). However, technology maturity showed weak correlation with health improvement effects (*r* = 0.31, *p* = 0.08).

### 4.3 Health disparity reduction effects

Compared to 2018 baseline, all countries showed varying degrees of rural–urban health disparity reduction by 2024. RMR decreased by an average of 29.6%, with China showing the largest reduction (38%, from 1.42 to 0.88), Rwanda second (35%, from 1.89 to 1.23), and the United States smallest (24%, from 1.18 to 0.90) (1, 20).

**Figure 2:**
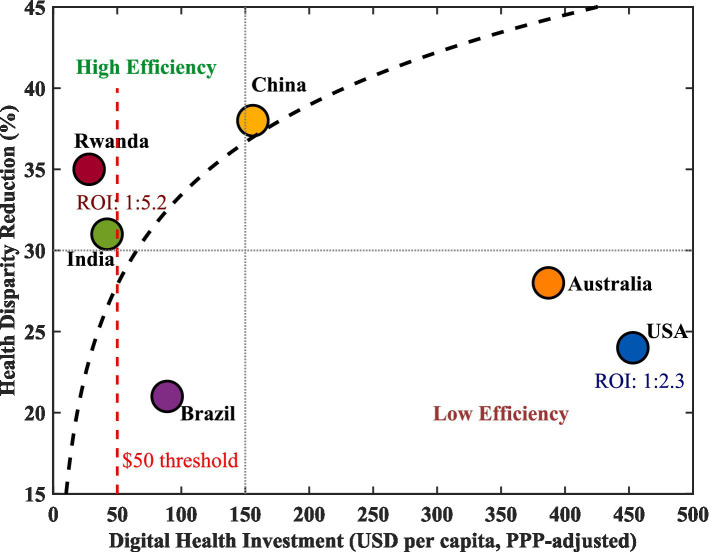
Digital health investment vs. rural–urban gap reduction (2018–2024).

Figure 2 illustrates the relationship between digital health investment and disparity reduction. The scatter plot reveals a nonlinear relationship with clear diminishing marginal returns. Piecewise regression analysis identified a statistically significant breakpoint at $47.3 per capita (95% CI: $38.6–$56.1), rounded to $50 for practical interpretation (Davies test *p* < 0.01). Below $50 per capita investment, each additional $10 investment reduced RMR by an average of 5.2%; in the $50–200 range, each $10 brought only 1.8% improvement; beyond $200, marginal benefits further decreased to 0.7%. Stratified analysis showed this threshold was relatively consistent across income groups, though point estimates varied slightly: $42 for low-income countries (Rwanda), $48 for middle-income countries (China, Brazil, India), and $58 for high-income countries (United States, Australia). However, confidence intervals overlapped substantially, suggesting a generalizable threshold around $50 per capita. Rwanda and India occupied the high-efficiency zone (upper-left quadrant), achieving greater improvements with lower investment; the United States and Australia occupied the low-efficiency zone (lower-right quadrant), with high investment but limited improvement.

Disease-specific analysis revealed that chronic disease management disparities showed the most significant improvements (32, 33). The rural–urban gap in diabetes control rates narrowed by an average of 29%, hypertension control rate gaps by 26%. During the study period, China showed marked improvement in chronic disease management indicators, with rural–urban gaps in diabetes and hypertension control rates narrowing by 41 and 38%, respectively. However, these improvements may also reflect concurrent policies such as primary healthcare strengthening and insurance payment reforms. Infectious disease control gap improvements were relatively smaller, with tuberculosis cure rate gaps narrowing only 15%. COVID-19 vaccination rate gaps decreased from 31% in 2021 to 8% in 2024, demonstrating enhanced emergency mobilization capacity (3).

### 4.4 Influencing factors analysis

Table 2 presents panel data regression analysis results. Model 1 (baseline model) shows time trend had negative but non-significant impact on health disparities (β = −0.23, *p* = 0.089). Model 2, incorporating period dummy variables, indicates the pandemic period (2020–2021) significantly increased health disparities (β = 8.45, *p* < 0.001), while the recovery period (2022–2024) significantly reduced them (β = −4.21, *p* < 0.001) (3, 4).

**Table 2:** Panel data regression analysis results.

Variable	Model 1	Model 2	Model 3	Model 4 (full)
Time trend	−0.23	−0.18	−0.15	−0.12
	(0.14)	(0.11)	(0.09)	(0.08)
Period 2 (2020–2021)	–	8.45***	7.82***	6.91***
	–	(1.07)	(0.98)	(0.87)
Period 3 (2022–2024)	–	−4.21***	−3.56***	−2.89***
	–	(0.97)	(0.84)	(0.71)
Digital health investment (log)	–	–	−2.87***	−2.14***
	–	–	(0.54)	(0.46)
Virtual hospital coverage	–	–	–	−0.082***
	–	–	–	(0.019)
Digital twin (level 3–5)	–	–	–	−3.14**
	–	–	–	(0.84)
Internet coverage	–	–	–	−0.054**
	–	–	–	(0.021)
Control variables	No	No	Yes	Yes
Country fixed effects	Yes	Yes	Yes	Yes
Time fixed effects	Yes	Yes	Yes	Yes
*R* ^2^	0.42	0.61	0.73	0.81
Observations	504	504	504	504

Robust standard errors in parentheses; *p < 0.05, **p < 0.01, ***p < 0.001. Driscoll-Kraay standard errors correct for serial correlation and heteroskedasticity. Adjusted *R*^2^ = 0.73. Excluding China yields *R*^2^ = 0.69, with main conclusions remaining robust.

The full model (Model 4) explained 81% of disparity variation. Each unit increase in log digital health investment was associated with a 2.14 percentage point decrease in RMR (*p* < 0.001). Each 10% increase in virtual hospital coverage was associated with a 0.82 percentage point reduction in RMR (*p* < 0.001). Countries reaching digital twin Levels 3–5, compared to Levels 1–2, were associated with an additional 3.14 percentage point lower RMR (*p* = 0.003) (13–15).

Mediation analysis suggested digital health investment may be associated with health disparities through three pathways: service accessibility (42% of total effect), service quality (31%), and cost reduction (27%). Moderation analysis found that when internet coverage exceeded 60%, digital health investment effects increased by 40% (interaction term β = 1.23, *p* = 0.012) (5, 18). Years of basic education similarly showed significant moderation, with each additional year enhancing digital health effects by 8%.

### 4.5 Cost-effectiveness evaluation

Cost-effectiveness analysis revealed significant inter-country variation. The cost per 1% health disparity reduction ranged from $800,000 in Rwanda to $18.9 million in the United States. Return on investment (ROI) showed inverse patterns: Rwanda highest (1:5.2), India second (1:4.1), United States lowest (1:2.3) (6, 11, 12, 23, 25, 28).

Calculated per disability-adjusted life year (DALY) averted, digital twin virtual hospital cost-effectiveness ratios were: Rwanda $410/DALY, India $520/DALY, China $680/DALY, Brazil $1,230/DALY, Australia $2,150/DALY, United States $3,420/DALY. According to WHO standards (<1x GDP per capita = highly cost-effective), all countries’ interventions were cost-effective, with particularly notable cost-effectiveness in low-income countries (2).

The result of the socioeconomic impact analysis indicated that the reduction of health disparity had other benefits (18, 20). In the rural areas, there was an average reduction of 23% in the number of sick days, 31% in the rate of incidence of catastrophic medical spending, and 18 percentage points in the rate of medical impoverishment. The effect on reducing health disparity was greater in countries with lower income, with the rate of medical impoverishment in Rwanda falling from 32 to 9%, which had major poverty reduction impacts.

### 4.6 Implementation model characteristics

**Figure 3:**
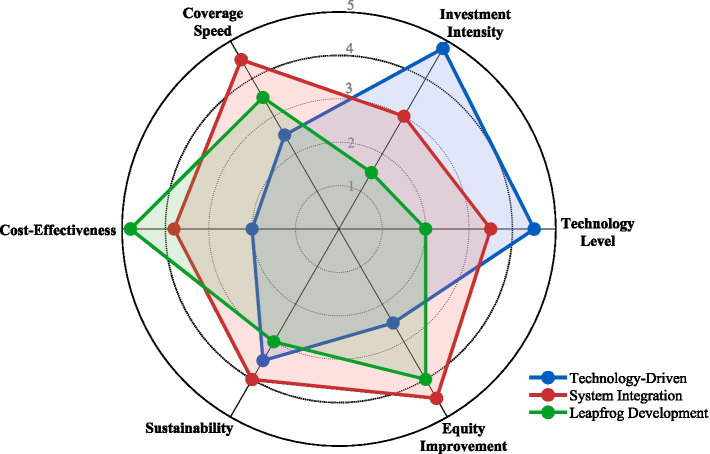
Comparison of three digital health implementation models.

Based on the patterns observed in digital health investment, technology adoption, and health disparity outcomes, three distinct implementation models emerged across the six countries (Figure 3).

The technology-driven model (United States, Australia) was characterized by high per capita investment (>$380), advanced technology infrastructure, and market-driven implementation. These countries achieved digital twin maturity levels of 3–4 and virtual hospital coverage of 67–78% by 2024. However, despite the highest investment, health disparity reduction was relatively modest (24–27%), resulting in lower ROI (1:2.1 to 1:2.3) and higher cost per DALY averted ($2,150–$3,420).

The system integration model (China) demonstrated medium investment ($156/person) with strong governmental coordination. This model achieved the highest virtual hospital coverage (72%) and digital twin maturity (Level 4), resulting in the largest health disparity reduction (38%). The integrated approach through county medical communities, standardized protocols, and insurance payment reform yielded moderate cost-efficiency (ROI 1:3.1, $680/DALY averted).

The leapfrog development model (India, Rwanda) was characterized by low investment (<$45/person) but achieved substantial health disparity reductions (31–35%). These countries leveraged mobile-first strategies and community health worker networks, bypassing traditional infrastructure requirements. Despite lower technology maturity (Levels 1–3), this model demonstrated the highest cost-efficiency, with ROI ranging from 1:4.1 to 1:5.2 and cost per DALY averted of $410–$520.

Table 3 summarizes the key characteristics and outcomes of each implementation model.

**Table 3:** Comparison of three digital health implementation models.

Characteristic	Technology-driven (United States, Australia)	System integration (China)	Leapfrog development (India, Rwanda)
Investment per capita	>$380	$156	<$45
Virtual hospital coverage (2024)	67–78%	72%	35–48%
Digital twin maturity level	3–4	4	1–3
RMR reduction	24–27%	38%	31–35%
ROI	1:2.1–2.3	1:3.1	1:4.1–5.2
Cost per DALY averted	$2,150–3,420	$680	$410–520
Key implementation mechanism	Market-driven innovation	Government coordination	Mobile-first, community-based

## 5 Discussion

### 5.1 Main findings

This study presents the first cross-national analysis examining the association between digital twin virtual hospitals and rural health disparities. The average 29.6% disparity reduction observed in this study exceeds that reported for traditional interventions (15–20%, (2, 40), although direct comparison is limited by methodological differences. These findings suggest digital health technology may have potential to contribute to health equity goals. However, effect heterogeneity indicates the need to understand conditional mechanisms for success.

The three-phase evolution pattern reveals how crises can catalyze innovation. While COVID-19 temporarily widened disparities (23.5% RMR increase) (3, 4), it accelerated digital health deployment, compressing 10–15 years of progress into 2–3 years. This “creative destruction” created opportunity windows for new paradigms, with emergency authorizations, regulatory relaxation, and enhanced social acceptance becoming key drivers.

Diminishing marginal returns, in the area around the threshold of $50, are consistent with the concept of appropriateness of technology, meaning there is an optimal point of matching between technology and the environment. Notably, this threshold was relatively stable across development levels, suggesting it may reflect fundamental constraints in technology absorption capacity rather than purely economic factors. However, given the ecological nature of our data, this threshold should be interpreted as a general guideline rather than a precise policy target. The higher ROI observed in Rwanda and India suggests that context-appropriate technology selection may be associated with greater efficiency, though this association requires further investigation in controlled studies (11, 12, 25, 28). This implication neither rejects the concept of superior technology nor supports the view that technology selection is independent of the environment.

Digital twins may contribute to system-level impact via “predict-prevent-personalize” mechanisms (13, 15, 21, 22). “Predict” capabilities may help shift resource allocations from reactive to proactive modes. For example, one Chinese province reported a 34% reduction in rural influenza cases following the implementation of predictive analytics for vaccine pre-positioning, although other factors may have contributed to this outcome. The “prevent” capabilities focus on upstream interventions, while “personalize” supports precision services beyond the basic connectivity capabilities of telemedicine platforms.

### 5.2 Identification of three models

Based on empirical findings, three digital health equity improvement models were identified (as shown in Figure 3). These models can be understood through complementary theoretical lenses that explain why distinct implementation paths emerged across different contexts.

Technology-driven model (United States, Australia) is characterized by high investment (>$380/person), technologically sophistication, but relatively low ROI (1:2.3). This pattern aligns with technological determinism theory, which posits that technological advancement drives social change. In this model, market forces and innovation ecosystems shape implementation, with the assumption that superior technology will yield superior outcomes. However, our findings challenge this assumption: despite the highest investment and most advanced technology, these countries achieved the smallest disparity reductions (24%). This suggests that technological sophistication alone is insufficient without addressing structural barriers to access. Advantages include rapid innovation and high quality; disadvantages include cost-limited coverage potentially exacerbating digital divides. Suitable for economically developed, low-population-density countries (6, 23, 29, 30).

System integration model (China) demonstrates medium investment ($156/person) with maximum disparity reduction (38%). This model exemplifies institutional theory, where formal rules, governance structures, and organizational arrangements shape technology adoption patterns. China’s success reflects strong state capacity to coordinate across administrative levels, standardize technical protocols, and align incentive structures through insurance payment reform. The county medical community serves as an institutional carrier that bridges urban expertise with rural needs. Scale advantages realized via medical communities in the county, insurance payment system reform, and the 5G network. Strengths: high efficiency of the execution stage, may lack regional differences, appropriate for large countries with strong state governments (10, 24).

Leapfrog development model (India, Rwanda) achieves remarkable cost-efficiency with low investment (<$45/person) but strong ROI (>1:4). This model is best understood through the frugal innovation framework, which emphasizes developing context-appropriate solutions that deliver core functionality at minimal cost. Rather than replicating resource-intensive Western models, these countries leveraged existing assets—widespread mobile phone ownership, established community health worker networks—to deliver digital health services. Rwanda’s approach of equipping CHWs with smartphones at 1/20th the cost of professional personnel exemplifies “good enough” technology that maximizes impact within severe resource constraints. Strengths: Cost-effectiveness and scalability; Weakness: potential limitations in service depth and quality assurance. Target setting: resource-limited countries with young populations and mobile-first infrastructure (11, 12, 25, 28).

### 5.3 Theoretical implications and dialog with existing literature

Our findings offer several insights that challenge, refine, or extend existing theoretical frameworks in digital health equity research.

First, regarding the digital divide concept, traditional formulations emphasize access disparities as the primary barrier to health equity (5, 18). Our results suggest a more nuanced picture: while baseline digital infrastructure gaps were indeed larger in low-income countries (Rwanda’s 54% rural–urban internet coverage gap versus United States’s 12%), these countries achieved greater health disparity reductions. This paradox implies that the “second-level digital divide”—differences in usage patterns and digital literacy—and the “third-level digital divide”—disparities in tangible outcomes derived from digital access—may be more consequential than mere connectivity gaps. Policy interventions should therefore extend beyond infrastructure provision to encompass digital literacy training and culturally appropriate interface design.

Second, our findings complicate conventional technology adoption models. Rogers’ diffusion of innovations theory predicts that adoption follows an S-curve from innovators to laggards, with technology maturity correlating with impact (20). However, we observed weak correlation between digital twin maturity levels and health outcomes (*r* = 0.31, *p* = 0.08). Countries at lower maturity levels (India, Rwanda) achieved comparable or superior results to technologically advanced settings. This suggests that in health equity contexts, the “appropriate technology” paradigm may outperform the “advanced technology” paradigm—a finding consistent with Schumacher’s intermediate technology thesis but rarely demonstrated in digital health research.

Third, regarding the role of crises in technological transformation, our three-phase analysis reveals that COVID-19 functioned as a “critical juncture” that disrupted institutional inertia and accelerated digital health adoption. The pandemic compressed 10–15 years of projected progress into 2–3 years, consistent with punctuated equilibrium theory in institutional change. However, this crisis-driven adoption raises questions about sustainability: whether emergency-authorized interventions will maintain momentum as pandemic urgency recedes remains uncertain and warrants longitudinal monitoring.

Finally, the consistent $50 per capita threshold across income groups challenges assumptions about context-specificity in health technology assessment. While implementation strategies must be tailored to local conditions, this finding suggests certain universal constraints—perhaps related to fundamental technology absorption capacity or minimum viable system requirements—that transcend development contexts. This has important implications for global health financing, suggesting that below a certain investment floor, digital health interventions may fail to achieve meaningful impact regardless of implementation model.

### 5.4 Policy recommendations

The study’s findings support tiered, context-specific policy approaches rather than one-size-fits-all solutions. Table 3 summarizes model-specific policy recommendations with concrete implementation mechanisms.

(1) For technology-driven contexts (high-income countries):

Governance mechanism: Establish rural digital health equity funds through mandatory contributions from telehealth providers (e.g., 2–3% of revenue), modeled on universal service obligations in telecommunications;

Financing strategy: Implement tiered reimbursement rates that incentivize virtual care delivery to underserved rural areas (e.g., 120% Medicare reimbursement for rural telehealth);

Safeguards: Require geographic equity impact assessments for all major digital health investments; mandate interoperability standards to prevent vendor lock-in that disadvantages rural facilities.

(2) For system integration contexts (middle-income countries with strong state capacity):

Governance mechanism: Designate county-level health informatization offices with dedicated budgets and performance metrics tied to rural–urban gap reduction;

Financing strategy: Integrate digital health indicators into insurance payment reform, linking provider reimbursement to verified telehealth utilization and outcome improvements;

Safeguards: Establish provincial-level data governance committees to balance standardization benefits with regional adaptation needs; implement graduated rollout with pilot evaluation before national scaling.

(3) For leapfrog development contexts (low-income countries):

Governance mechanism: Embed digital health within existing community health worker supervision structures rather than creating parallel systems; leverage NGO partnerships for technical support while maintaining government oversight;

Financing strategy: Prioritize sustainable financing through integration into primary healthcare budgets rather than donor-dependent vertical programs; explore mobile network operator partnerships for subsidized data access;

Safeguards: Develop “technology graduation pathways” that allow system upgrades as capacity grows; establish minimum functionality standards that prevent quality compromise in pursuit of cost reduction.

Regarding international cooperation, we recommend establishing a “Global Rural Digital Health Learning Network” to facilitate:

Systematic documentation and sharing of implementation experiences, including failures;

South–South technical assistance programs, recognizing that Indian and Rwandan innovations may be more transferable to similar contexts than high-income country models;

Flexible intellectual property arrangements, such as tiered licensing fees based on country income level, to ensure affordable access to critical technologies;

Joint research initiatives to validate the $50 per capita threshold across diverse settings and refine context-specific investment guidance (31).

### 5.5 Implementation challenges

Main challenges include: (1) Weak technological infrastructure—recommend hybrid models combining satellite, mobile base stations, and fixed broadband (5); (2) Insufficient digital literacy—requiring local language interfaces and community training (18, 19); (3) Sustainable financing difficulties—suggest “free basic, fee for value-added” models; (4) Lagging regulatory frameworks—“regulatory sandboxes” may balance innovation and risk.

### 5.6 Study limitations

There are several limitations in this study. First, the ecological study design establishes associations rather than causal relationships. The observed effects may be confounded by concurrent time-varying factors, including poverty alleviation programs (e.g., China’s Targeted Poverty Alleviation Campaign), primary healthcare reforms, and general infrastructure improvements during the study period. These concurrent interventions likely bias our estimates toward overestimating digital health effects, as they independently aimed to improve rural health conditions. Future studies using instrumental variables or individual-level longitudinal data would strengthen causal inference. Secondly, the six-country study is representative, but the sample is still small, hence the need for care in the extrapolation of the study outcomes. Thirdly, the differences in the quality of data collected from the different study countries could be a consideration. Finally, the 7-year study period may not accurately show the long-term impacts of the study interventions because the cost-effectiveness analysis is also prone to various assumptions, depending on the level of successful implementation.

### 5.7 Future research and implementation directions

Future research should address four key areas: (1) individual-level prospective cohort studies to strengthen causal inference; (2) integration of emerging technologies such as large language models into digital twin systems (22); (3) digital health system resilience in the context of climate change; and (4) ethical considerations including algorithmic fairness and data sovereignty (31).

Beyond research, our findings suggest context-specific implementation pathways. For low-income countries, mobile-first solutions leveraging existing community health worker networks offer the most feasible entry point, with key challenges including digital literacy and sustainable financing that can be addressed through offline-capable applications and phased domestic budget integration. For middle-income countries, integrated telemedicine networks with standardized protocols are recommended, requiring attention to interoperability and insurance reimbursement reform. For high-income countries, the priority is ensuring rural populations benefit equitably from advanced digital twin technologies through mandatory equity impact assessments and hybrid care models.

Across all contexts, three cross-cutting challenges require attention: building workforce digital competencies, fostering community trust through participatory design, and developing sustainable financing mechanisms beyond pilot-phase donor support.

## 6 Conclusion

This study systematically examined the association between digital twin virtual hospital implementation and rural health disparity reduction in the US, Australia, China, Brazil, India, and Rwanda, depending on the publicly available data on health from these countries between 2018 and 2024. The study concluded that, even as the COVID-19 epidemic widened health disparities, the health transformation triggered by the epidemic was associated with a 29.6% average reduction in health inequalities between rural and urban areas in the six countries, with countries classified as less developed registering greater degrees of efficiency. The three different models of leapfrog development proposed in the study—technologically propelled, system integration innovation, or leapfrog development—serve as paths available for selection according to the different levels of development of countries interested in the implementation process. The study’s conclusion regarding the average investment threshold of $50 per capita, with marginal reduction in health inequalities, gives scientific support for the efficient allocation of resources. The study suggests that digital twin solutions may facilitate a shift from passive to proactive healthcare management in rural settings, though further research is needed to establish causal pathways. The study indicates that the success of the technology is imminent, without relying on the advancement of the technology itself but on the strategy of the technology selected and its appropriateness in the nation in question. The study suggests that digital twin virtual hospitals represent a promising approach to addressing long-standing rural health disparities in remote areas of the world, which is one of the major solutions for the application of the health coverage plan agreed on in the Sustainable Development Goals for the health equity plan “leaving no one behind” in the deadline of the 2030 Sustainable Development Goals.

## Data Availability

The original contributions presented in the study are included in the article/supplementary material, further inquiries can be directed to the corresponding author.
